# A Study of the Methane Oxidation Mechanism and Reaction Pathways Using Reactive Molecular Simulation and Nonlinear Manifold Learning

**DOI:** 10.1021/acsomega.4c07094

**Published:** 2024-10-17

**Authors:** Jiang Wang, Jiaxuan Tang, Fuye Chen

**Affiliations:** College of Science, Guizhou Institute of Technology, Boshi Road, Dangwu Town, Gui’an New District, Guizhou 550025, China

## Abstract

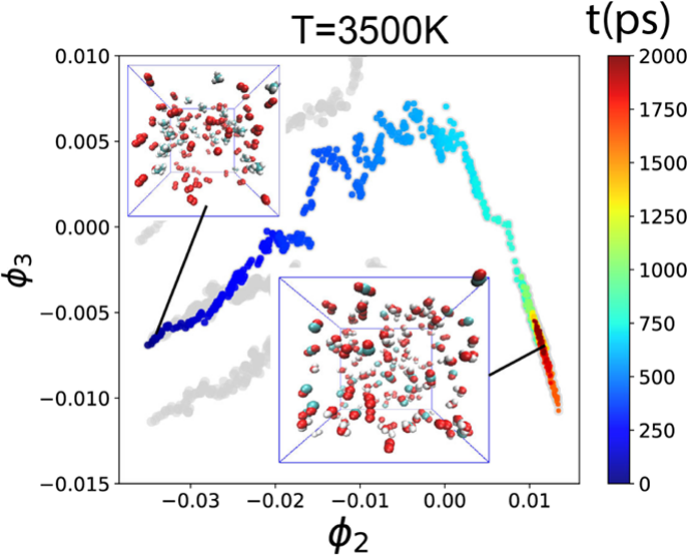

Methane, as the primary component of natural gas, is a vital energy resource extensively utilized through oxidation reactions. These reactions yield diverse radicals and molecules via varying intermediate reaction routes, contingent upon the oxidation conditions. In this study, we employ reactive molecular dynamics simulations to investigate the early-stage mechanism of methane oxidation across different temperatures and methane/oxygen conditions. Our analysis reveals distinct variations in species count, initial reaction times, and the spectrum of the main reactions/molecules under diverse conditions. Notably, both full oxidation of methane (FOM) and partial oxidation of methane (POM) are observed in all simulations, with FOM favored under high-temperature and fuel-lean conditions, while POM prevails in low-temperature and fuel-rich environments. Furthermore, we utilize nonlinear manifold learning techniques to extract a 2D manifold from the reaction state space, identifying two collective variables governing the reaction pathways. This research provides a systematic understanding of the initial stage mechanisms of methane oxidation under varying conditions, offering useful insights into chemical science and fuel engineering.

## 1 Introduction

As global energy demands continue to rise, natural gas and shale gas have emerged as primary sources of fuel due to their abundant reserves and comparatively cleaner emissions compared to traditional fossil fuels.^1−4^ Composed mainly of methane (CH_4_), both natural gas and shale gas play pivotal roles in meeting energy needs. The full oxidation of methane (FOM) in oxygen is represented by the equation
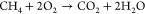
1and releases energy of Δ*H* = −891 kJ/mol under the standard condition.^5,6^ Under some specific conditions, the partial oxidation of methane (POM) will take place, it follows the reaction of
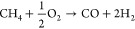
2which releases less energy, but it is highly efficient and rapid, yielding H_2_ as a byproduct. Hydrogen (H2) holds promise as a competitor for future green energy solutions.^7,8^ The adiabatic flame temperature of methane combustion typically falls within the range of 1800–2200 K.^9^ However, in the presence of pure oxygen, this temperature can skyrocket to as high as 3900 K.^10^

Methane oxidation is a complex process characterized by multiple substeps and the generation of numerous radicals. Even simplified representations, like the complete oxidation of methane (FOM) or partial oxidation of methane (POM) equations, involve a plethora of intermediate reactions.^11−13^ The GRI-Mech 3.0 model, a widely used methane oxidation mechanism, incorporates 53 species and 253 different reactions based on experimental analysis.^5^ To streamline computational efforts, simplified models have been proposed to coarse-grain the reaction mechanism,^14,15^ such as an 18-reaction and 14-species model by Peters et al.^16^ Additionally, under varying combustion conditions, such as temperature and concentration, the species and reactions involved can differ significantly.^5,17,18^ Despite extensive research on methane oxidation, there remains a need to explore further details at the atomistic level, particularly regarding the characteristics of the reaction pathways and how these pathways are influenced by varying reaction conditions.

Experiments serve as a direct means to study methane oxidation,^19−21^ yet they often struggle to provide atomistic-level insights into reaction processes, such as reaction routes and radical generation. In the realm of computation, quantum mechanical (QM) approaches offer accurate insights and detailed reaction mechanism of small chemical systems.^22−24^ Moreover, classical molecular dynamics simulations (MD) utilize Newtonian mechanics to model atom interactions, enabling simulations of systems containing millions of atoms over microseconds. However, classical MD lacks the capability to simulate chemical reactions due to its inability to represent bond formation and breaking. To address this limitation, density functional-based tight binding (DFTB), which is an approximation of the DFT, is used to handle large reactive systems that are more accurate than classical MD but less computationally expensive than full DFT.^25−27^ In another hand, reactive force fields in MD have been developed.^28−32^ Notably, the ReaxFF force field, proposed by Van Duin et al., accurately captures bond dynamics in MD simulations while maintaining computational efficiency.^33−36^ ReaxFF has emerged as one of the most widely used reactive force fields, applied in various studies of chemical reactions, including fuel combustion^37−41^ and hydrocarbon pyrolysis.^42−44^ In this article, we employ ReaxFF to investigate methane oxidation under diverse conditions.

Several recent studies employed ReaxFF to investigate various aspects of methane oxidation. Page, Gan, and their colleagues examined the low-temperature partial oxidation of methane, leading to the production of H_2_ and acetylene.^45,46^ He et al. explored the full oxidation of methane under explosive conditions, observing the formation of a large number of free radicals in a methane/oxygen mixture with a density of 0.2184 g/cm^3^ and temperatures up to 3000 K.^47^ Methane oxidation, often in conjunction with other components such as H_2_O and CO_2_, has been widely investigated across multiple research groups, typically within the temperature range of 2400–3600 K.^48−51^ Sun et al. explored electric field-assisted methane combustion, observing how the electric field influenced reaction pathways.^51^ Wang et al. utilized ReaxFF to investigate the initial stage mechanism of soot formation during methane combustion, revealing that radicals play a crucial role in accelerating soot formation.^52^

Methane combustion simulations yield trajectory data in a multidimensional species space. If *N*_s_ species are generated during methane oxidation, the species space dimensionality is *D* = *N*_s_. Given that methane oxidation can produce over a hundred types of molecules and radicals, the corresponding species space dimensionality exceeds one hundred. Effectively analyzing such high-dimensional data is essential for extracting useful information from simulations, and machine learning offers a promising approach for this task.

Machine learning (ML) offers many powerful tools for data analysis, encompassing both supervised and unsupervised learning techniques.^53−55^ In recent years, ML has found widespread application in biology and chemistry for analyzing complex high-dimensional data.^56−59^ For instance, AI-guided approaches have been employed for the rational design of functional polymers or proteins,^60,61^ deep learning techniques have been utilized for coarse-graining MD force fields,^62−64^ and ML methods have been applied to solve Schrödinger equations.^65^ Moreover, machine learning has been instrumental in enhancing the accuracy of reactive force fields such as ReaxFF.^66^

Diffusion maps (dMaps) represent an unsupervised machine learning technique, specifically a nonlinear manifold learning method aimed at dimensionality reduction. Its primary objective is to extract the intrinsic low-dimensional nonlinear manifold from high-dimensional data,^67−69^ thereby enabling subsequent data analysis or visualization. Illustrated in Figure 2, dMaps are capable of extracting the intrinsic 2D manifold from a curved Swiss-roll structure from a 3D Euclidean space. Widely utilized, dMaps have been instrumental in extracting low-dimensional free energy surfaces in protein folding and self-assembling of colloids or asphaltene systems.^70−72^

In this study, we perform ReaxFF simulations to explore methane combustion in oxygen at various temperatures and concentrations. Subsequently, we analyze the initial reaction properties and utilize diffusion maps (dMaps) to extract low-dimensional manifolds and discern reaction pathways from the high-dimensional species space. This approach aims to offer a more straightforward and clear understanding of the initial reaction mechanism. This research provides a systematic understanding of the initial stage mechanisms of methane oxidation under varying conditions, offering useful insights for chemical science and fuel engineering.

## 2 Methods

### 2.1 Reactive Molecular Dynamics Simulation

**Figure 1:**
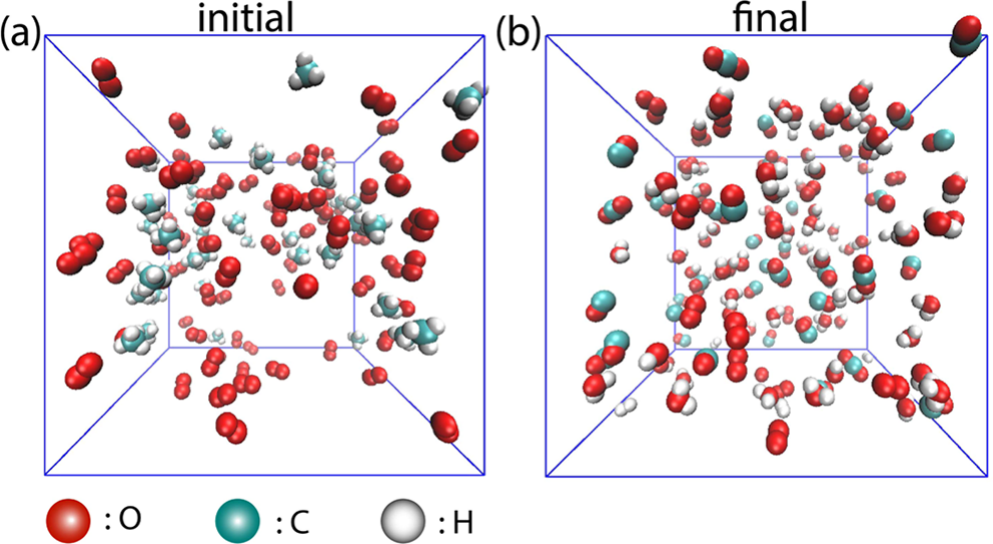
Snapshots of the S0 simulation, where *T* = 3500 K, box side length *L* = 3.5 nm, and initial *n*(CH_4_):*n*(O_2_) = 1:2. (a) Initial configuration and (b) final snapshot after 2000 ps.

In this research, we employ molecular dynamics simulations to investigate methane oxidation at different temperatures and concentrations. The first simulation (S0) consists of 40 CH_4_ molecules and 80 O_2_ molecules, in the stoichiometric ratio of 1:2 as per eq 1. The simulation is conducted within a periodic box measuring 3.5 × 3.5 × 3.5 nm^3^, and the temperature is 3500 K. Initially, CH_4_ and O_2_ molecules are randomly distributed within the box. Figure 1a illustrates the initial configuration of S0, while Figure 1b depicts the final state of the simulation, where only CH_4_ and O_2_ are visible in Figure 1a, whereas CO_2_, CO, H_2_, H_2_O, and H_2_O_2_ are observed in Figure 1b.

It is worth noting that while realistic methane combustion temperatures typically fall below 2200 K and occur within a few seconds, our ReaxFF simulations, along with many others in the field, employ higher temperatures. This choice is made because elevated temperatures facilitate rapid reactions that occur within a few picoseconds, allowing them to be captured by molecular dynamics simulations. Despite the disparity in time and temperature between the ReaxFF simulations and experiments, the initial reaction mechanisms and kinetics observed in high-temperature ReaxFF simulations demonstrate good agreement with experimental data. This approach has been successfully utilized by numerous other researchers.^33,48,73^

In this research, we explored five systems under different conditions, labeled as S0 through S4. Systems S1 and S2 are characterized by lower temperatures compared to S0. Conversely, systems S3 and S4 feature varying concentrations of CH_4_ and O_2_: S3 represents a fuel-lean condition with a ratio of *n*(CH_4_):*n*(O_2_) = 1:4, while S4 represents a fuel-rich condition with a ratio of *n*(CH_4_):*n*(O_2_) = 3:2, where *n*(•) denotes the number of molecules in the simulation box. Table 1 summarizes the parameters for all of the systems.

**Table 1:** Parameters Set Up for Each Simulation, Bold Texts Correspond to Varied Parameters That Are Different from S0

ID	*n*(CH_4_)	*n*(O_2_)	*n*(CH_4_):*n*(O_2_)	*T* (K)	*L* (nm)
S0	40	80	1:2	3500	3.5
S1	40	80	1:2	**3000**	3.5
S2	40	80	1:2	**2600**	3.5
S3	**24**	**96**	**1:4**	3500	3.5
S4	**72**	**48**	**3:2**	3500	3.5

The Large-scale Atomic/Molecular Massively Parallel Simulator (LAMMPS) package^74,75^ was employed to conduct MD simulations in this study. For all simulations, the ReaxFF force field and CHO-2016 parameter sets developed by van Duin et al.^34^ were utilized. In ReaxFF, the potential energy of the system (*E*_system_) is determined by the bond order, which is a function of the distance between a pair of atoms. The expression for *E*_system_ is given by
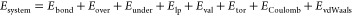
3where the first six *E* terms on the right-hand side represent bond energy, overcoordinate energy penalty, undercoordination stability, long pair energy, valence angle energy, and torsion angle energy. These terms are related to the bond forming and breaking. The last two terms (*E*_Coulomb_, *E*_vdWaals_) represent electrostatic and van der Waals potentials.^34,35^

Each simulation begins with an equilibration phase in the canonical (NVT) ensemble at a temperature of 300 K for 100 ps. Subsequently, the temperature is raised from 300 K to the target temperature within 100 ps, during which time the reaction is turned off to prevent methane oxidation. The production run phase employs the canonical (NVT) ensemble with a simulation time step of 0.1 fs. Each simulation runs for 2000 ps on 4 × 5.2 GHz Intel i9–12900KF cores. Temperature control is achieved using a Nosé–Hoover thermostat with a damping parameter of 0.01 ps, and charge equilibrium is performed at every step.^76,77^ Molecular species are identified using the built-in algorithm in LAMMPS, and frames are saved every 1 fs, resulting in 2 × 10^6^ frames in each simulation trajectory. A bond order cutoff of 0.3 is employed to identify molecular or radical species during the simulation. To reduce the statistical uncertainty, three independent simulations are performed for each condition listed in Table 1, and physical quantities are calculated by averaging over these three simulations. A typical simulation trajectory from S0 is available in the Supporting Information (SI) as a video file. Simulation input script and coordinate files (stoichiometric, fuel-lean, fuel-rich) are also provided in the SI.

It is worth noting that as a classical simulation approach, ReaxFF can provide qualitatively correct results effectively. To improve simulation accuracy, QM-based approaches, such as Car–Parrinello molecular dynamics (CPMD), can be considered.^78,79^ Moreover, after obtaining basic information about key reaction routes from ReaxFF simulations, the accurate reaction energy barriers of these identified routes can be further calculated using Density Functional Theory (DFT),^80,81^ and the reaction rates can then be calculated using variational transition state theory (VTST).^82−86^ Thus, ReaxFF simulation could serve as a valuable and efficient first-step exploration of the studied reaction process.

### 2.2 Nonlinear Manifold Learning

In this research, we employ diffusion maps as a nonlinear dimensionality reduction tool to extract the intrinsic low-dimensional reaction pathways from the high-dimensional species space. A total of 207 different types of molecules (including radicals) are created from 5 simulations, forming a species basis denoted as
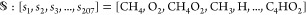
The complete species list can be found in the SI. For a frame in the simulation, its state can be characterized by a 207-dimensional species number vector *N⃗* = [*n*_1_, *n*_2_, ···, *n*_207_], where *n*_*i*_ is the number of molecules of type *s*_*i*_ found in the frame. However, this number vector *N⃗* is not normalized because different molecules *s*_*i*_ may contain different numbers of atoms, and ∑_*i* = 1_^207^*n*_*i*_ is not a constant throughout the simulation. To normalize the state vector, and notice that each system has a conserved number of atoms, we can then define a normalized state vector: each component *v*_*i*_ of the normalized state vector *V⃗* = [*v*_1_, *v*_2_, ···, *v*_207_] is defined as , where *m*_*i*_ is the number of atoms in one molecule of type *s*_*i*_, for example, *s*_1_ = CH_4_, *s*_2_ = O_2_, and *m*_1=5_, *m*_2=2_, etc. *A* = ∑_*i* = 1_^207^(*n*_*i*_ · *m*_*i*_) is the normalization factor representing the total number of atoms in the system so that *v*_*i*_ represents the ratio of atoms fall into type *s*_*i*_. In this definition, *V⃗* is normalized such that ∑_*i* = 1_^207^*v*_*i*_ = 1, and *V⃗* will be used in the diffusion maps calculation.

**Figure 2:**
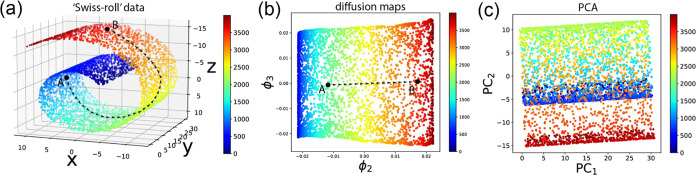
A toy model shows the difference between diffusion maps and the PCA. (a) Original data are in a intrinsically 2-dimensional nonlinear “Swiss-roll” structure, and lie in a 3-dimensional Euclidean space. Path connecting points A and B lies in the nonlinear manifold. (b) dMaps could extract the 2D intrinsic manifold and span it by top eigenvectors. (c) Traditional linear dimensionality reduction approach, such as the principal component analysis (PCA) fails to extract the nonlinear manifold, as can be seen that there are still many overlapping points in the space spanned by top PCs.

Diffusion maps (dMaps), proposed by Coifman et al.,^68,87−89^ model the data as a random walk or diffusion process on an intrinsic low-dimensional manifold. This manifold can be nonlinear and curved, embedded within a high-dimensional Euclidean space, as illustrated in Figure 2a, where data points diffuse in the intrinsic 2D nonlinear Swiss-roll manifold within a 3D Euclidean space. The dMaps algorithm extracts the intrinsic dimensions corresponding to the slowest diffusion modes, as depicted in Figure 2b. The slowest diffusion mode from point A to point B follows the arrow-dashed path in the Swiss-roll nonlinear manifold. In this regard, dMaps outperforms regular linear approaches such as principal component analysis (PCA), as shown in Figure 2c. Previously, both we and others have applied dMaps to various MD simulation data, including protein and alkane folding simulations, as well as the self-assembly of asphaltene and patchy colloid particles.^58,70−72,90−94^

In this research, we will utilize diffusion maps (dMaps) to extract low-dimensional reaction pathways from the 207-dimensional state space of methane oxidation data. The process involves several steps. First, we compute the pairwise distance *d*_*ij*_ between each pair of frames *i*, *j*, where *i*, *j* = 1, 2, 3, ···,*N*, and *N* are the number of frames from the simulation trajectory. We use the Euclidean distance between state vectors *V⃗* of frames *i* and *j* as the pairwise distance: *d*_*ij*_ = |*V⃗*_*i*_ – *V⃗*_*j*_|.

Next, we obtain the hopping probability matrix **A** by applying a Gaussian kernel to the distance matrix: *A*_*ij*_ = exp(−*d*_*ij*_^2^/2ϵ), where ϵ is the bandwidth.^68,87−89^ This operation assigns large hopping probabilities to adjacent pairs with small distances (*d*_*ij*_ → 0 ⇒ *A*_*ij*_ → 1), while distant pairs have negligible probabilities (*d*_*ij*_ → ∞ ⇒ *A*_*ij*_ → 0).

In the third step, we derive the Markov matrix **M** using **M** = **D**^–1^**A**, where **D** is a diagonal normalization matrix, representing the row sum of **A**: *D*_*ii*_ = ∑_*k* = 1_^*N*^*A*_*ik*_.

Finally, we perform an eigen decomposition of the Markov matrix **M** to obtain a series of eigenvalues [λ_1_, λ_2_, ···,λ_*N*_] ∈ (0, 1] in descending order, along with associated eigenvectors [**ϕ**_1_, **ϕ**_2_, ···, and **ϕ**_*N*_]. Note that the Markov matrix **M** is a *N* × *N* matrix, and eigenvector **ϕ**_*i*_ has *N* components. Eigenvectors corresponding to large eigenvalues represent the slowest diffusion modes and are the most important intrinsic dimensions. Notably, the first eigenvalue, λ_1_ = 1, and its corresponding eigenvector **ϕ**_1_ are disregarded, as they represent the steady state.

For dimensionality reduction, each high-dimensional state point is projected onto the low-dimensional space spanned by the top *k* eigenvectors. For instance, the *i*-th frame from the simulation is represented in a *k* – 1-dimensional coordinate: state_i_ → [**ϕ**_2_(*i*), **ϕ**_3_(*i*), ···, **ϕ**_*k*_(*i*)], where **ϕ**_*k*_(*i*) denotes the *i*-th component of the *k*-th eigenvector **ϕ**_*k*_. When plotting the eigenvalues in descending order [λ_1_, λ_2_, ···, λ_*N*_*C*__] ∈ (0, 1], as shown in Figure 4a, we observe the trend in their magnitudes, referred to as the eigenvalue spectrum. A gap in the spectrum indicates a significant drop in value, implying that the indices on the left-hand side of the gap are more important due to their larger eigenvalues compared with the smaller ones on the right-hand side. The selection of *k* is determined by the position of the gap in the eigenvalue spectrum. Usually, *k* = 2 or 3 is enough to capture meaningful patterns and information on the manifold.

It is necessary to note that this is the first time we have applied dMaps to the reaction species space to extract low-dimensional representations. The methane oxidation reaction, although simple, is not trivial and serves as a prototype to verify this application. For methane oxidation, it is possible to analyze the reaction process quantitatively using traditional calculations, which can be compared with the results from dMaps. Once the application of dMaps to methane oxidation is validated, we can extend its use to more complex reactions. While traditional analysis only focuses on the calculation of specific well-defined quantities, dMaps will allow us to perform dimensional reduction and obtain a clear and straightforward collective description of reaction paths. Moreover, dMaps is not limited to be applied to ReaxFF simulation trajectories, but can also be applied to the QM-based simulations, such as trajectories from CPMD.^78,79^

## 3 Results and Discussion

### 3.1 Methane Oxidation Mechanism

**Figure 3:**
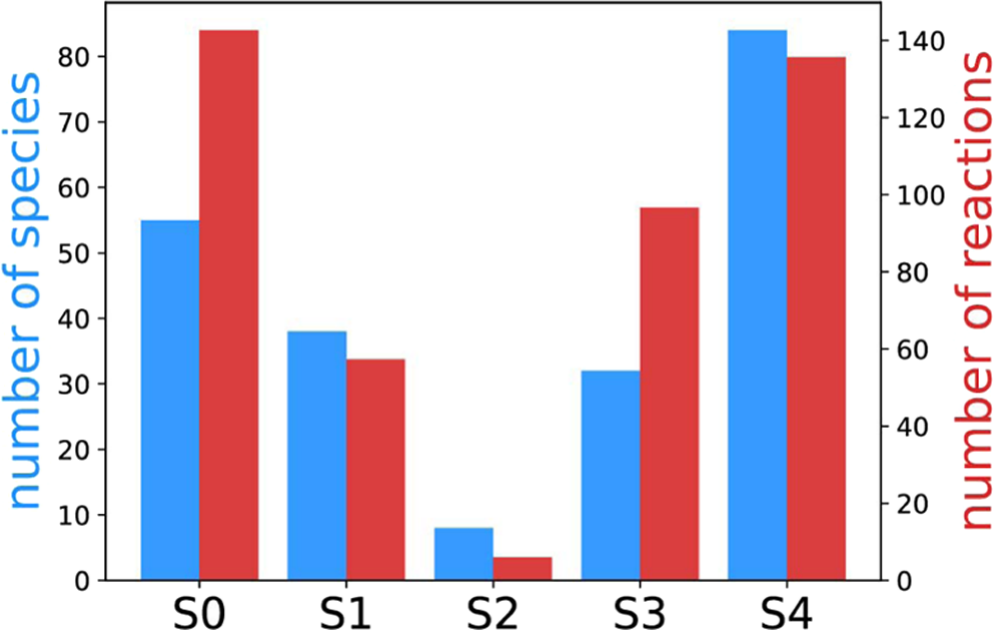
Number of all species and reaction types appeared during each simulation.

**Figure 4:**
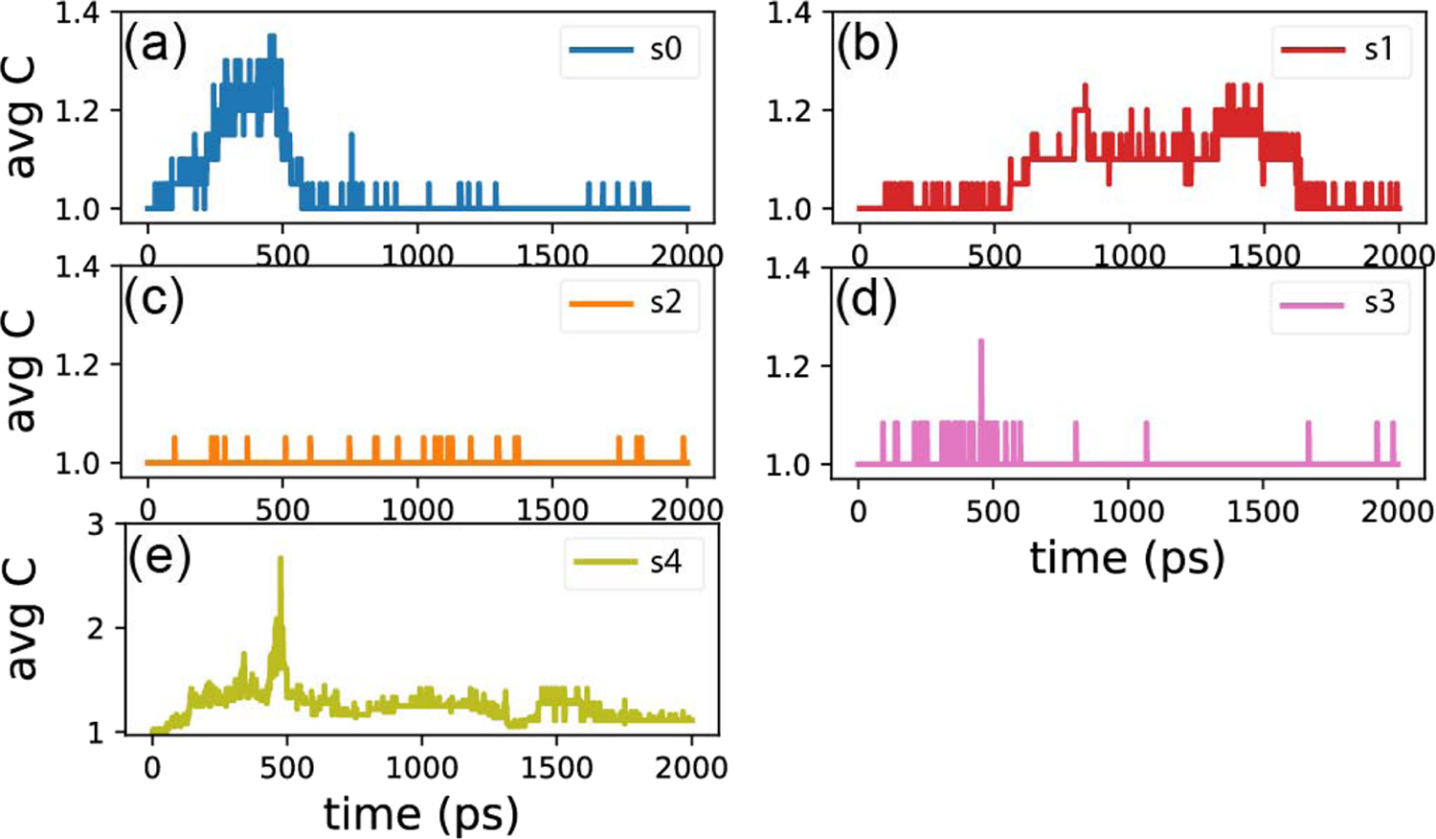
Average carbon number in carbon compounds as a function of time in each simulation for (a) S0, (b) S1, (c) S2, (d) S3, and (e) S4.

Under different conditions, the number of observed species of molecules and radicals, as well as the number of reaction types, vary. As depicted in Figure 3a, the blue bars represent the total number of species observed in each simulation, and the red bars denote the number of reaction types. It is evident that both the number of species and reaction types decrease as the temperature decreases. Notably, simulation S4, conducted under fuel-rich conditions, exhibits the highest number of species compared to other conditions. This is attributed to the greater concentration of carbon atoms in S4, which are more prone to forming larger and more diverse compounds. This trend is further illustrated in Figure 4, where panels a–e showcase the average carbon number in carbon compounds as a function of time for each simulation. In panel e, representing S4, the size of carbon compounds is notably higher compared to S3 and other conditions, leading to a larger number of observed species.

**Figure 5:**
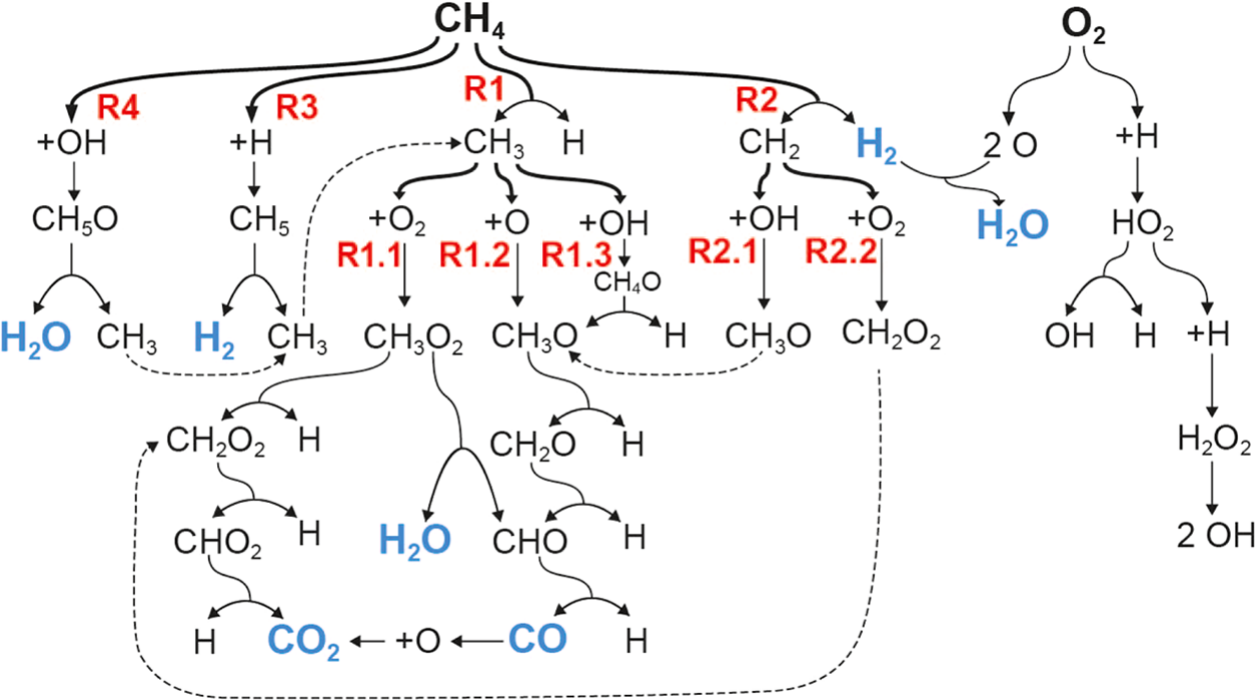
Initial key reaction routes network. Bold text corresponds to the reactant, blue text indicates the final products, and red number indicates key reactions. Dashed lines connect same radicals to each other.

From the 5 simulations, a total of 207 distinct molecular and radical species are observed, with the number of species differing for each simulation and being less than 207. Among these, six molecules
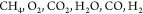
and eight radicals
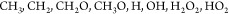
are particularly prevalent across all simulations. Although each simulation encompasses hundreds of intermediate reactions, certain reactions are notably more prominent than others, forming the primary initial reaction route network depicted in Figure 5.

The disassociation of CH_4_ involves four main reactions (4–12), with reactions 4 and 8 each leading to three and two significant secondary reactions, respectively (5–7, 9–10). These nine typical reactions are detailed in Table 2. Notably, different conditions favor particular molecules, radicals, and reactions over others, a topic that will be further explored in subsequent sections. To assess the accuracy of the ReaxFF simulations, we calculated the reaction potential energies for typical reactions—4, 6, 8, 11, and 12—using DFT and compared them with the ReaxFF results. While the reaction energies from ReaxFF are not exactly identical to those from DFT (see Table S1 in the SI), they generally show good agreement, with relative errors below 30%.

**Table 2:** Initial Key Reactions

reaction ID	reaction
R1	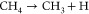 R1
R1.1	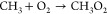 R1.1
R1.2	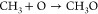 R1.2
R1.3	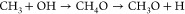 R1.3
R2	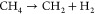 R2
R2.1	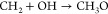 R2.1
R2.2	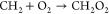 R2.2
R3	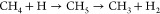 R3
R4	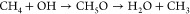 R4

The initial reaction mechanisms differ across the 5 simulations. To quantify this, we calculated the time taken for the system to consume 10% of the methane molecules, as depicted in Table 3. In simulations S0–S2, we observe that the initial time increases as the temperature decreases. For instance, at *T* = 3500 K, the initial time is a mere 31.72 ps, whereas at *T* = 2600 K, it takes 1581 ps for 10% of the methane to disassociate. The notably shorter initial times for S4 suggest that fuel-rich conditions are conducive to initializing the reaction.

**Table 3:** Time Used to Consume Initial 10% CH_4_ in Each Simulation

condition	S0	S1	S2	S3	S4
time (ps)	31.72	179.15	1581.19	57.91	21.49

### 3.2 2D dMaps of Methane Oxidation Pathways

**Figure 6:**
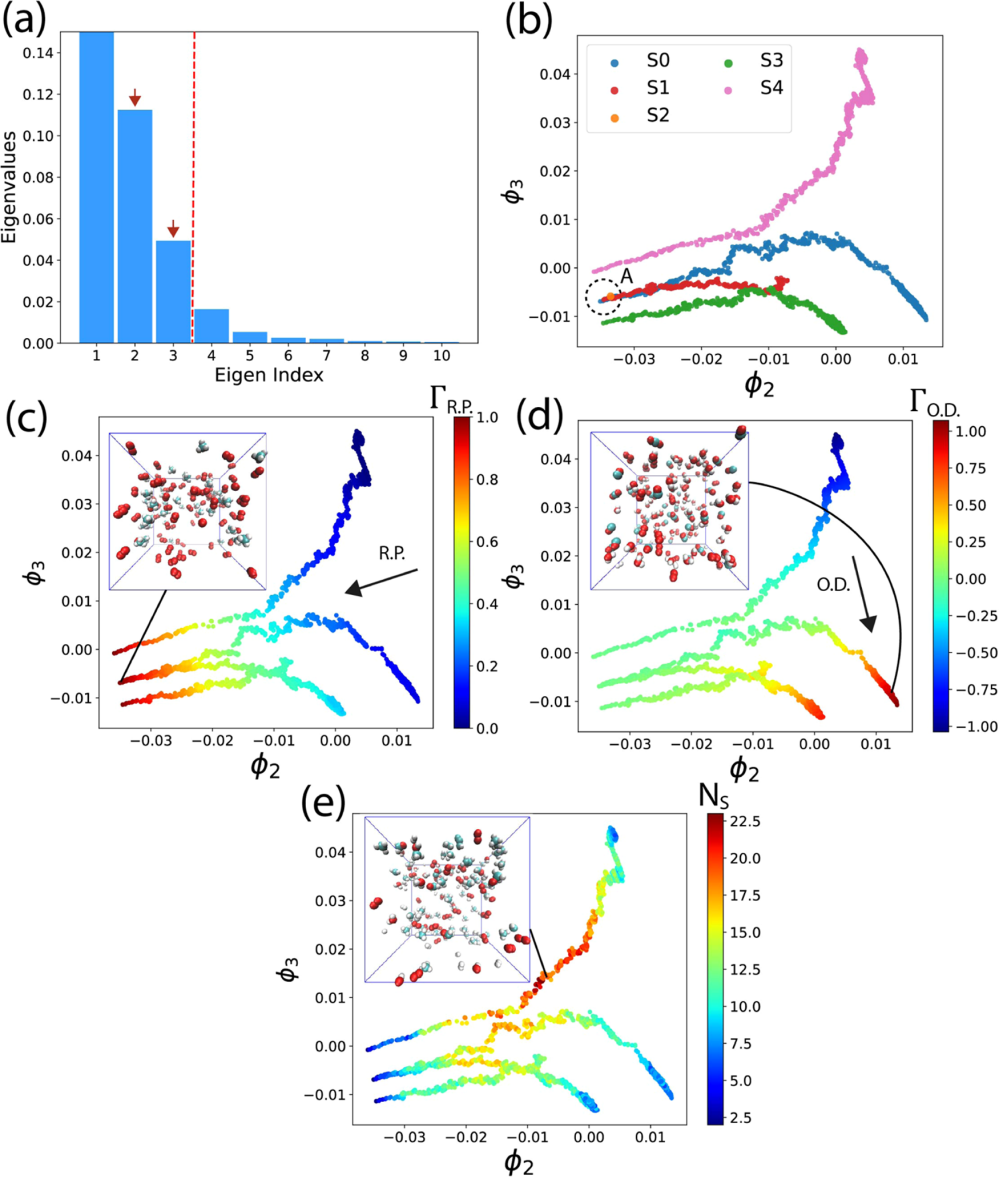
(a) Eigenvalue spectrum of dMaps in the descending order, a gap can be identified between the third and the fourth eigenvalues, meaning that the intrinsic dimensionality is 2 (eigenindexes 2 and 3). (b) All data points are plotted in the 2D dMaps, and each simulation is colored differently. Dashed circle A is the initial state. (c) All points are colored with the reactant proportion Γ_R.P._. (d) All points are colored with oxidation degree Γ_O.D._. (e) All points are colored with the number of species.

We utilize dMaps on the composite data gathered from the 5 simulations. This approach enables all of the data to be represented using the same eigenvectors, facilitating comparisons across different simulations.^92,95^ The spectrum of the 10 largest eigenvalues is depicted in Figure 6a. Notably, the first eigenvalue λ_1_ is 1, and λ_3_ is significantly larger than λ_4_, suggesting that the data can be effectively characterized using two dimensions spanned by **ϕ**_2_ and **ϕ**_3_ (indicated by the red vertical dashed line). Although we could include more indices for analysis in higher-dimensional space, this would introduce additional complexities. Therefore, we will focus only on the top two eigenvectors.

Figure 6b illustrates the 2D dMaps of all state points derived from 5 simulations, with each simulation represented by a distinct color. Notably, each simulation manifests a 1-dimensional structure, indicative of different reaction pathways. Over time, the state points progress from left to right along each pathway, as evident in Figures 9 and 12, where the points are color-coded based on time. Since simulations S3 and S4 feature varying initial *n*(CH_4_):*n*(O_2_) ratios, they exhibit distinct pathways and starting points. Conversely, simulations S0–S2 share the same initial CH_4_ to O_2_ ratio, resulting in a shared starting point (denoted by dashed circle A in Figure 6b), but ultimately culminate at different locations along diverse pathways. It is worth noting that the points corresponding to the S2 condition are clustered at the starting location. This occurs because, under the low temperature in S2, the reaction produces only a few species, making the system remain similar to its original state throughout the 2000 ps simulation.

Eigenvectors **ϕ**_2_ and **ϕ**_3_ represent collective variables that are nonlinear functions of the system’s degrees of freedom. However, these variables lack apparent physical interpretation. To elucidate the meaning of each collective variable, we introduce two functions: Γ_R.P._, the reactant proportion, and Γ_O.D._, the oxidation degree. These functions are used to color each point in Figure 6c,d, and Γ_R.P._ is defined as
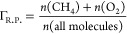
4

The function Γ_R.P._ quantifies the reaction progression by representing the proportion of reactants (methane and oxygen) in the system, with Γ_R.P._ constrained within the range [0, 1]. Initially, at the start of the simulation, Γ_R.P._ equals 1.0. However, as time elapses and more reactants are consumed, Γ_R.P._ gradually decreases.

The black arrow in Figure 6c illustrates the trend direction in which Γ_R.P._ increases the fastest (the gradient of Γ_R.P._). We can see that Γ_R.P._ is negatively correlated with **ϕ**_2_ more strongly than **ϕ**_3_. In the 2D dMaps, the bottom left region corresponds to the raw system, where minimal oxidation reactions have occurred. As depicted in the inset snapshot, an abundance of CH_4_ and O_2_ molecules is present in the simulation box. As state points transition from left to right, the values of **ϕ**_2_ increase, indicative of greater consumption of CH_4_ and O_2_, resulting in smaller Γ_R.P._ values.

Figure 6d shows the 2D dMaps with points being colored with the oxidation degree (Γ_O. D._), which is defined as
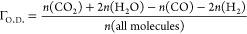
5

As outlined in eqs 1 and 2, the full oxidation of methane (FOM) equation signifies that one CH_4_ molecule yields one CO_2_ and two H_2_O molecules, while in the partial oxidation of methane (POM), one CH_4_ produces one CO and two H_2_ molecules. Γ_O.D._ is computed by subtracting the products of the POM equation from those of the FOM equation. Therefore, Γ_O.D._ serves to illustrate the disparity between FOM and POM; a higher Γ_O.D._ value indicates the prevalence of FOM over POM.

The black arrow in Figure 6d represents the gradient of Γ_O.D._, and it is evident that Γ_O.D._ exhibits a stronger negative correlation with **ϕ**_3_ than the correlation with **ϕ**_2_, which is positive and weaker. Points located at the bottom right of the dMaps diagram (where **ϕ**_3_ is small yet Γ_O.D._ is large) represent states where FOM predominates, whereas small Γ_O.D._ values for points at the upper region (where **ϕ**_3_ is large) indicate a prevalence of POM over FOM. The inset snapshot of Figure 6d illustrates the presence of CO_2_, CO, H_2_, and H_2_O molecules, with H_2_O being relatively more abundant than the others at the end of the reaction pathway for S0, resulting in a large Γ_O.D._ value.

We note that the arrows in Figure 6c,d are almost perpendicular to each other. This indicates that the two quantities we identified, Γ_R.P._ and Γ_O.D._, can effectively serve as the normal basis to span the 2D manifold (**ϕ**_2_, **ϕ**_3_) discovered using dMaps.

In Figure 6e, each state point is colored according to the number of species (*N*_S_). Initially, located at the bottom left of the figure, *N*_S_ is minimal and approaches 2, as all simulations initially contain only methane and oxygen. As the simulations progress, the number of species gradually increases, peaking at the center of the dMaps diagram. Toward the end of the simulations, *N*_S_ decreases once more. During the final stages, intermediate molecules and radicals are progressively depleted, with final products, such as CO_2_, H_2_O, H_2_, and CO dominating the system. The inset of Figure 6e presents a representative snapshot characterized by a large *N*_S_. Here, the presence of polymers with multiple carbon atoms is noticeable, contributing to increased compound complexity and a richer diversity of molecules.

### 3.3 Temperature Effects on Methane Oxidation

**Figure 7:**
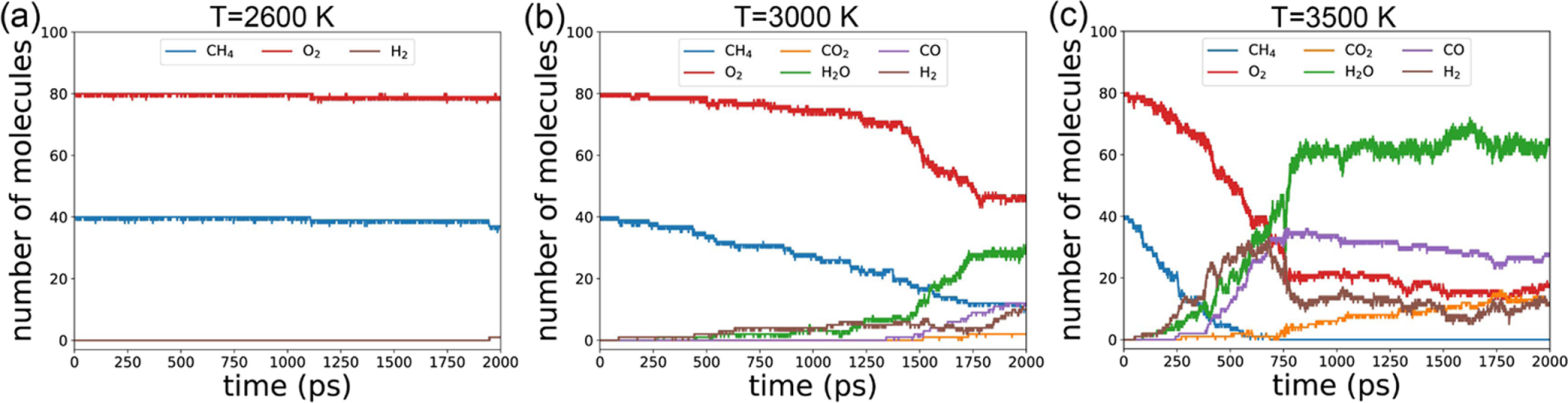
Number of molecules as a function of time for main products under different temperatures: (a) *T* = 2600 K, (b) *T* = 3000 K, (c) *T* = 3500 K.

We investigated the impact of temperature on methane oxidation, showcasing the evolution of the six primary molecules over time in Figure 7. At a lower temperature of *T* = 2600 K (Figure 7a), the quantities of CH_4_ and O_2_ remained nearly constant throughout the 2000 ps simulation. Only a few reactions occur toward the final stages, yielding a small quantity of H_2_. At this temperature, the production of final products and radicals is constrained, with details on the main radical numbers provided in Figure S1 in the SI.

With increasing temperature, as depicted in Figure 7b,c, methane and oxygen consumption accelerates, accompanied by the generation of more products over time. Notably, at *T* = 3500 K, CH_4_ is entirely depleted by 700 ps, while residual O_2_ remains despite the initial stoichiometric ratio of 1:2 for methane and oxygen. This observation suggests the occurrence of both the full oxidation of methane (FOM) and partial oxidation of methane (POM), leading to the formation of both CO_2_ and CO.

**Figure 8:**
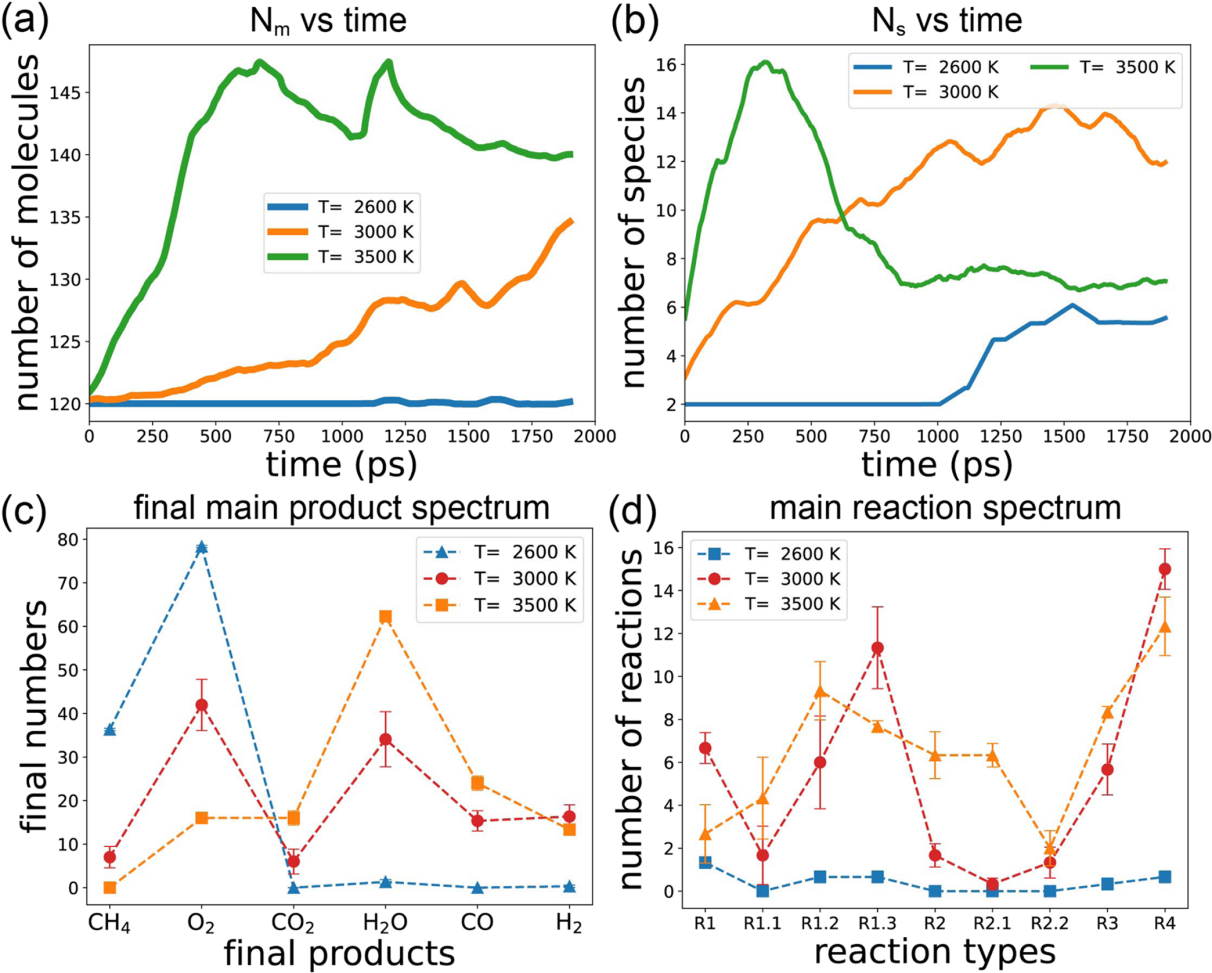
(a) Number of all molecules as a function of time, (b) number of species as a function of time, (c) final main product spectrum, and (d) main reaction spectrum at different temperatures.

The evolution of the total number of molecules (*N*_m_) and species (*N*_S_) over time is illustrated in Figure 8a,b, respectively. At *T* = 2600 K (Figure 8a), the system initially comprises 120 molecules (40 methane and 80 oxygen), with negligible variation observed throughout the simulation. Upon increasing the temperature to *T* = 3000 K, *N*_m_ gradually increases over time but fails to reach a maximum. Conversely, at *T* = 3500 K, *N*_m_ exhibits a more rapid ascent to the maximum value before gradually declining.

Figure 8b depicts the species count (*N*_S_) over time. At *T* = 2600 K, *N*_S_ remains at 2 for the first 1000 ps, after which additional species emerge, predominantly radicals and intermediate molecules, rather than dominant products. With *T* = 3000 K, *N*_S_ steadily rises until it peaks near the end of the 2000 ps simulation. For *T* = 3500 K, *N*_S_ exhibits rapid initial growth, followed by a sharp decline, stabilizing at approximately 7–8 for the remainder of the simulation.

Figure 8c illustrates the main product spectrum in the final state of each simulation. At *T* = 2600 K, only CH_4_ and O_2_ are present. As the temperature rises, the quantities of CH_4_ and O_2_ decrease while those of CO_2_, H_2_O, CO, and H_2_ increase. In Figure 8d, we observe the frequency of each typical reaction throughout the simulation. At *T* = 2600 K, the number of reaction types are minimal. As the temperature increases, all reaction counts rise, showing similar trends except for R2 (CH_4_ → CH_2_ + H_2_) and R2.1 (CH_2_ + OH → CH_3_O). Higher temperatures favor these two reactions disproportionately. We observe that the reaction count for R4 is the highest at both temperatures, which is consistent with previous DFT research by Zhu et al.^47^ In their study, they found that the primary reaction in the methane oxidation under stoichiometric conditions is CH_4_ → ^•^CH_3_, induced by ^•^OH radicals. This reaction corresponds to the R4 route in our reaction network. The second primary step involves the conversion from ^•^CH_3_ to CH_3_O^•^, which corresponds to the R1.2+R1.3 routes, and both R1.2 and R1.3 exhibit high reaction counts in Figure 8d. This consistency indicates that ReaxFF simulation of the methane oxidation could provide qualitatively correct results compared to more accurate DFT calculations.

**Figure 9:**
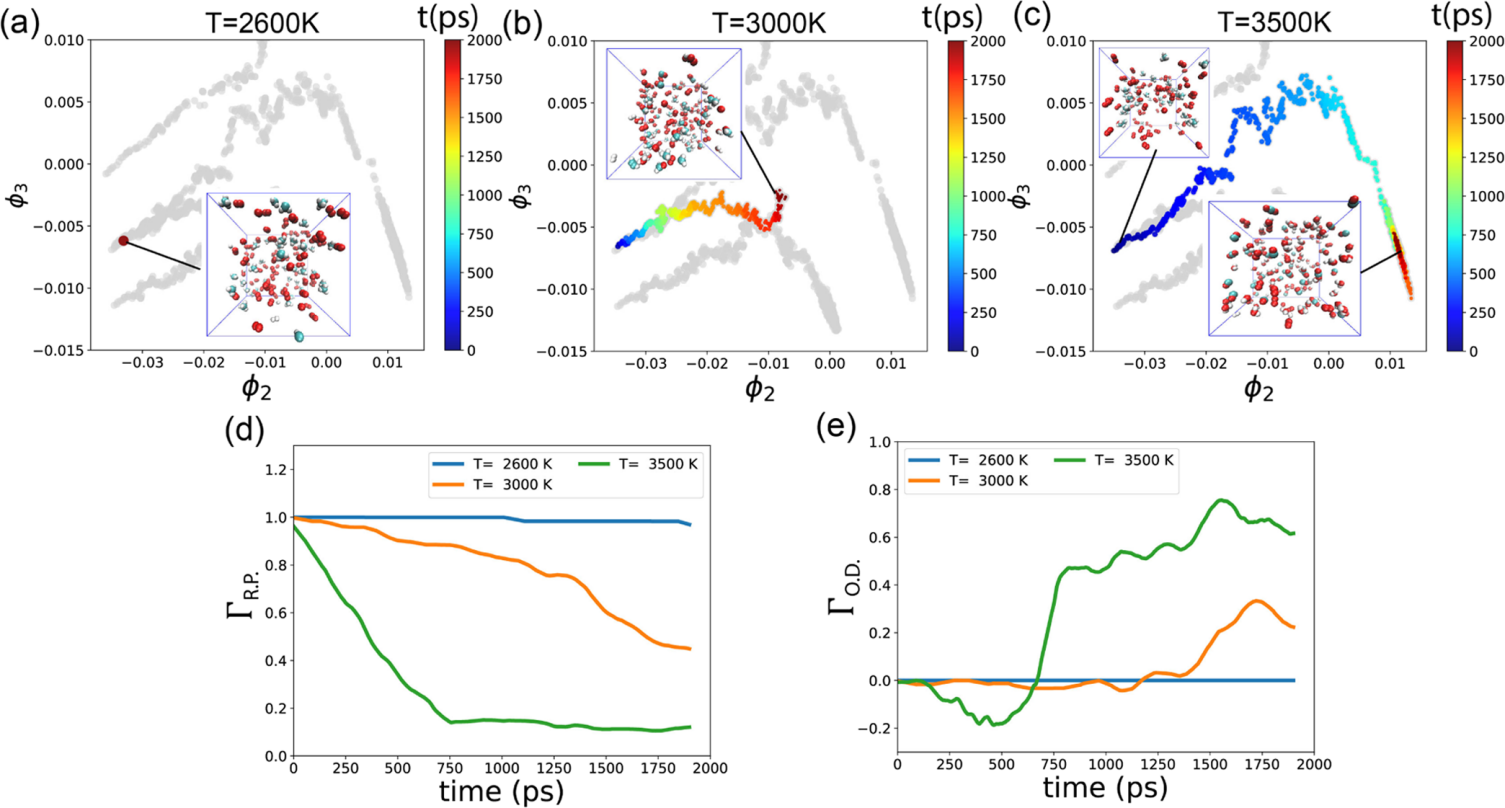
(a–c) Reaction pathways in 2D dMaps for different temperatures; points are colored with time (0–2000 ps). (d) Γ_R.P._ (reactant proportion) and (e) Γ_O.D._ (oxidation degree) as functions of time for different temperatures.

As depicted in Figure 9, we segregate data points from different simulations and plot them separately in dMaps. Each point is colored according to its corresponding time (ranging from 0 to 2000 ps). In Section 3.2, we determined that the intrinsic dimensionality of reaction paths is 2 and that Γ_R. P._ and Γ_O.D._ effectively explain the 2D data. Therefore, we plot the values of Γ_R.P._ and Γ_O.D._ for different temperatures over time, as shown in Figure 9e,f. The Γ_R.P._ and Γ_O.D._ curves are obtained by applying a moving window average with a window size of 100 ps so that the curve ends at *t* = 1900 ps.

In Figure 9a, at *T* = 2600 K, the data points are tightly clustered together, indicating that the final and initial states are closely situated. This occurs because there is a minimal change in state during the simulation at lower temperatures. The inset snapshot in Figure 9a illustrates the final state of the system, where CH_4_ and O_2_ still dominate despite the presence of a few H_2_. Throughout the simulation, the Γ_R.P._ values remain close to 1.0, while Γ_O.D._ consistently stays at 0.0.

As depicted in Figure 9d, with increasing temperature, the curve exhibits a steeper slope, indicating an increase in the reaction rate. This acceleration leads to a faster consumption of CH_4_ and O_2_ at higher temperatures. Concurrently, in Figure 9b,c, the data points gradually shift away from their initial positions, illustrating distinct reaction pathways corresponding to different temperatures.

When *T* = 3000 K, the depicted pathway appears shorter, with state points converging toward the center of the diagram. Notably, all data points exhibit similar **ϕ**_3_ and Γ_O.D._ values (Figure 9e), with Γ_O.D._ hovering around 0.0. As discussed in Section 3.2, this equilibrium indicates a competitive balance between FOM and POM, with both processes occurring at similar rates. Consequently, Γ_O.D._ values remain relatively stable, except for a slight increase during the final 500 ps, indicating a shift toward greater predominance of FOM over POM. The inset in Figure 9b offers a clear depiction of CO_2_, H_2_O, CO, and H_2_, reinforcing this interpretation.

When *T* = 3500 K, Γ_R.P._ experiences a rapid decline, as illustrated in Figure 9d, indicating an accelerated consumption of CH_4_ and O_2_ compared with lower temperatures. Notably, between 250 and 750 ps, state points in dMaps ascend to an upper location with **ϕ**_3_ = 0.007 (Figure 9c). Here, Γ_O.D._ values are small and approximately −0.2 (Figure 9e), suggesting a prevalence of POM over FOM during this period. As the simulation progresses, data points regress to the bottom right region of the dMaps diagram, where Γ_O.D._ values are large, indicating that FOM predominates over POM. The bottom inset snapshot in Figure 9c clearly illustrates the presence of CO_2_ and H_2_O, products of FOM.

The analysis using dMaps and classical calculations indicates that dMaps can serve as an initial heuristic tool to identify the most important collective properties of the reaction process (e.g., the two collective variables **ϕ**_2_ and **ϕ**_3_ in the methane oxidation system). This can then guide further quantitative calculations such as Γ_R.P._ and Γ_O.D._ in this study.

### 3.4 Concentration Effects on Methane Oxidation

**Figure 10:**
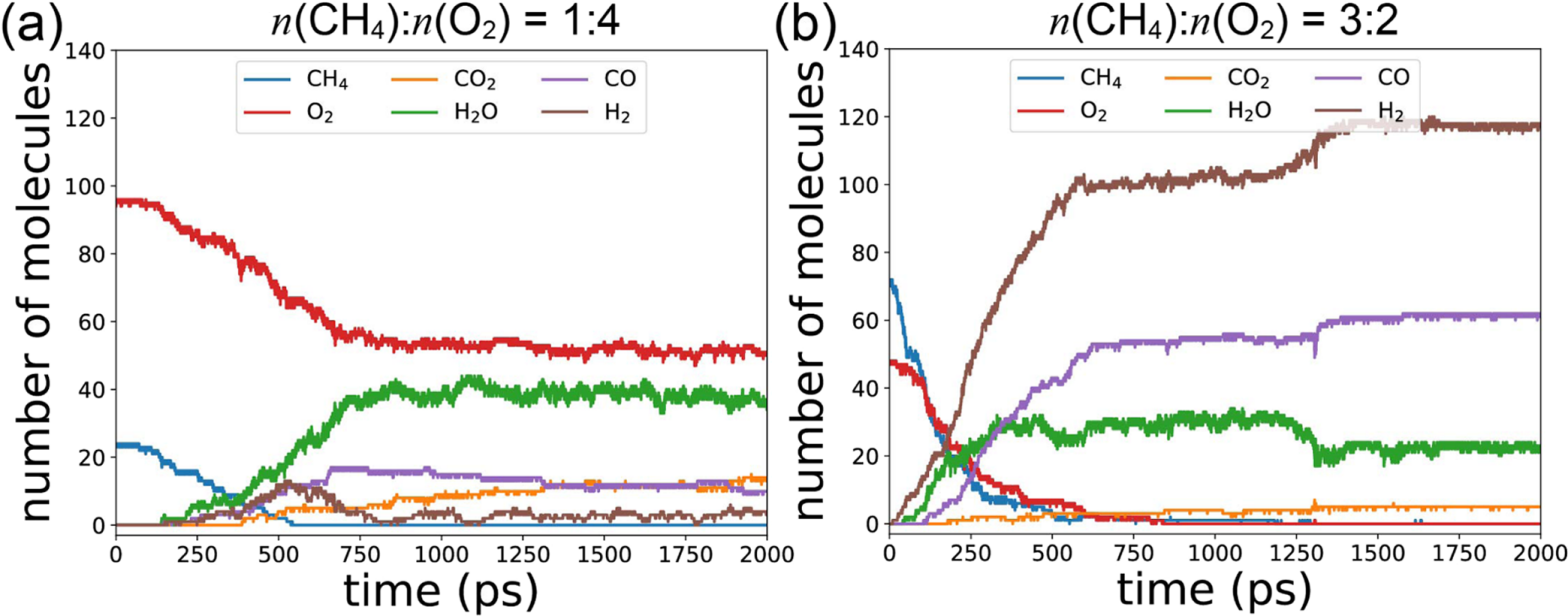
Number of molecules as a function of time for main products. (a) Fuel-lean condition with *n*(CH_4_):*n*(O_2_) = 1:4, (b) fuel-rich condition with *n*(CH_4_):*n*(O_2_) = 3:2.

Three different *n*(CH_4_):n(O_2_) ratios are explored: the fuel-lean condition with a ratio of 1:4, the stoichiometric condition with a ratio of 1:2, and the fuel-rich condition with a ratio of 3:2. Main molecule numbers as functions of time are displayed in Figure 10a,b for the fuel-lean and fuel-rich conditions, respectively. Results from the stoichiometric conditions are depicted in Figure 7c.

In Figure 10a, it is evident that all CH_4_ is consumed, while a significant portion of the remaining O_2_ remains. Conversely, in Figure 10b, both CH_4_ and O_2_ are entirely consumed. Comparing Figure 10a,b, we observe that the fuel-lean condition favors FOM, as evidenced by the elevated numbers of H_2_O and CO_2_. In contrast, in the fuel-rich condition, POM reactions predominate, with the numbers of CO and H_2_ surpassing those of other species, as depicted in Figure 10b.

**Figure 11:**
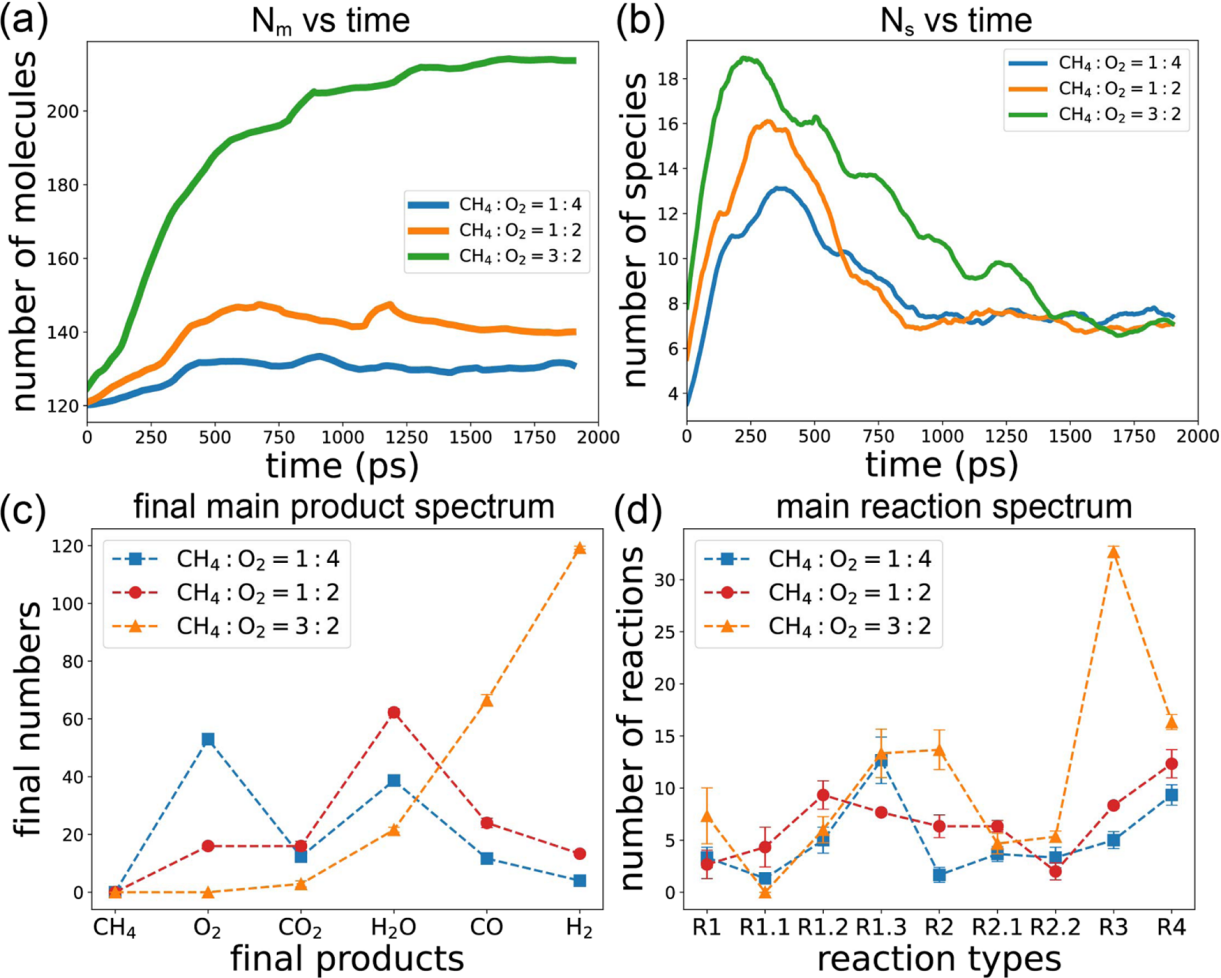
(a) Number of all molecules as a function of time, (b) number of species as a function of time, (c) final main product spectrum, and (d) main reaction spectrum with different *n*(CH_4_):*n*(O_2_) ratios.

*N*_*m*_ as a function of time for different *n*(CH_4_):*n*(O_2_) ratios is illustrated in Figure 11a. It is evident that *N*_*m*_ starts at 120 °C and increases over time, eventually reaching a plateau. As the *n*(CH_4_):*n*(O_2_) ratio rises, the plateau height also increases. Figure 11b depicts species number *N*_*S*_ as a function of time. A larger *n*(CH_4_):*n*(O_2_) ratio corresponds to a higher *N*_*S*_. This suggests that fuel-rich conditions are more conducive to generating both a greater quantity and a wider variety of molecules. As shown in Figure 4e, in the fuel-rich condition, the average carbon number in compounds is the highest among all conditions. Consequently, a greater diversity of allotropes and molecular structures is produced, leading to the highest *N*_*S*_ value.

The final product spectrum under different *n*(CH_4_):*n*(O_2_) ratios is depicted in Figure 11c. In the fuel-lean condition (*n*(CH_4_):*n*(O_2_) = 1:4), there are still many O_2_ molecules remaining after the 2000 ps simulation. In the stoichiometric condition (*n*(CH_4_):*n*(O_2_) = 1:2), the remaining oxygen decreases but does not reach zero, with a significant production of H_2_O molecules. Conversely, in the fuel-rich condition (*n*(CH_4_):*n*(O_2_) = 3:2), all CH_4_ and O_2_ are consumed, with suppressed production of H_2_O and CO_2_, while CO and H_2_ production is significantly enhanced. This indicates that the fuel-rich conditions favor POM reactions, whereas the fuel-lean condition favors FOM.

The spectrum of the reaction numbers is shown in Figure 11d. We observe that in the fuel-lean condition, reaction 8 (CH_4_ → CH_2_ + H_2_) is suppressed, while in the fuel-rich condition, both R2 and R3 (CH_4_ + H → CH_5_ → CH_3_ + H_2_) are enhanced.

**Figure 12:**
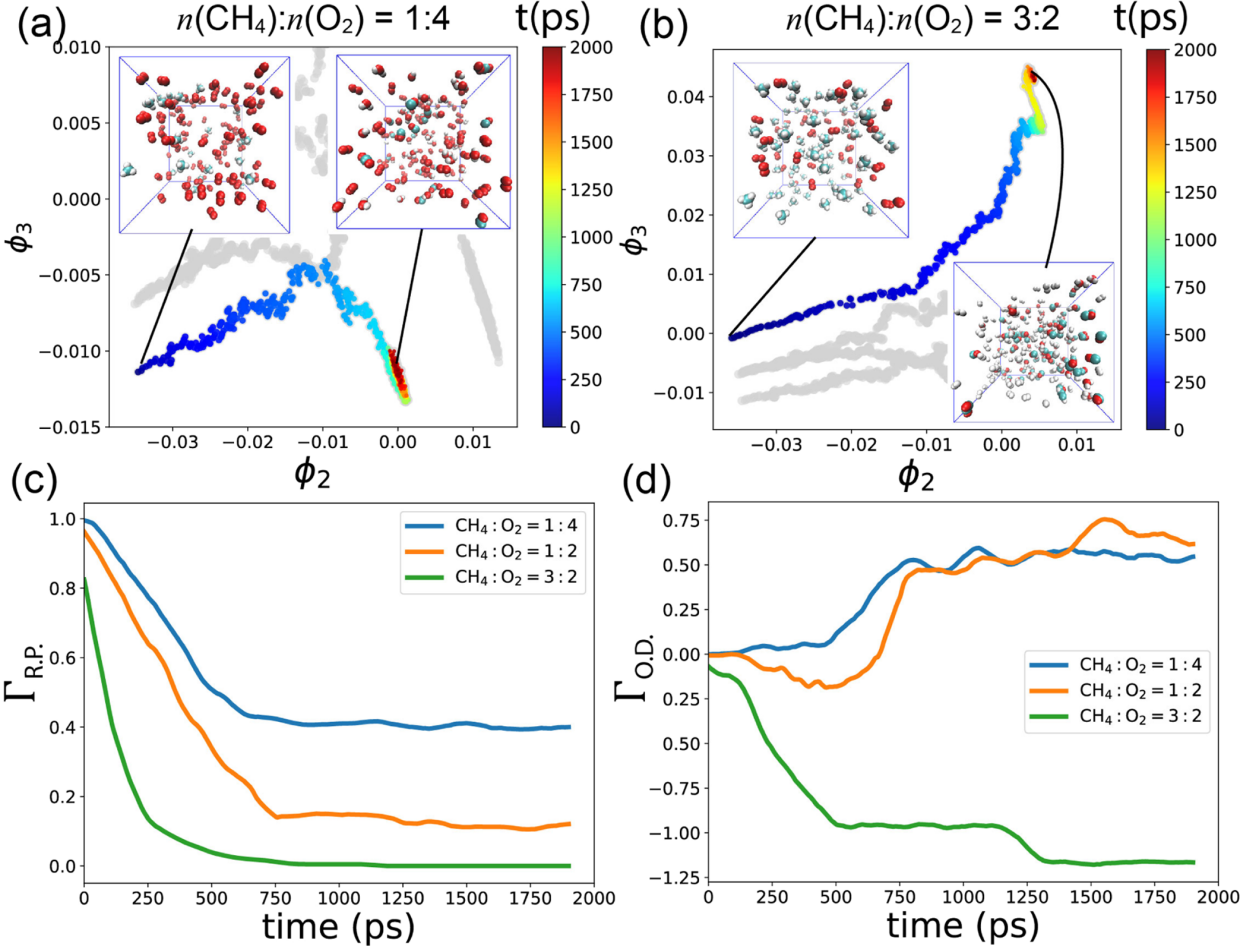
Reaction pathways in 2D dMaps, in (a) fuel-lean condition where *n*(CH_4_):*n*(O_2_) = 1:4, and (b) fuel-rich condition where *n*(CH_4_):*n*(O_2_) = 3:2, points are colored with time (0–2000 ps). (c) Γ_R.P._ (reactant proportion) and (d) Γ_O.D._ (oxidation degree) as a function of time for different *n*(CH_4_):*n*(O_2_) ratios.

The 2D reaction pathways under fuel-lean and fuel-rich conditions, spanned by **ϕ**_2_ and **ϕ**_3_, are depicted in Figure 12a,b, respectively. Figure 12c,d displays Γ_R.P._ and Γ_O.D._ values as functions of time. In Figure 12c, we observe that the reactants are quickly consumed and then reach plateaus for all three simulations. However, the consumption rate is higher when the concentration of methane is higher.

Together with the stoichiometric condition in Figure 9c, we observe that different *n*(CH_4_):*n*(O_2_) ratios result in distinct reaction pathways, with varying starting and ending locations. In Figure 12a, the reaction path for the fuel-lean condition curves downward, indicating small **ϕ**_3_ values, and ends with large Γ_O.D._ values (Figure 12d), suggesting a preference for FOM. The relatively short length of this path results in smaller **ϕ**_2_ but relatively larger Γ_R.P._ values compared to S0 and S4, indicating incomplete consumption of reactants. The snapshot inset in Figure 12a shows that many O_2_ molecules remain in the final state.

Conversely, in the fuel-rich condition shown in Figure 12b,d, the reaction path curves upward in the dMaps and ends with large **ϕ**_3_ but small Γ_O.D._ values, indicating a preference for POM. The snapshot inset at the bottom of Figure 12b illustrates the creation of numerous H_2_ and CO. The small Γ_R.P._ values at the end of this path suggest complete consumption of methane and oxygen in the fuel-rich condition.

## 4 Conclusions

In this study, we utilized reactive molecular dynamics simulations to investigate the initial mechanism of methane oxidation in pure oxygen across varying temperatures and reactant concentration conditions. Our analysis revealed several key findings:

Identification of the main products and reactions: We identified six primary products and nine key reactions across all of the simulations. Importantly, simulations conducted under different conditions yielded distinct product and reaction spectra.

Impact of conditions on reaction properties: Various aspects of the reactions, including initial reaction times, total numbers of species generated, and diversity of reaction types, were found to be dependent on the specific conditions of the simulations.

Extraction of 2-dimensional reaction pathways: Using the dMaps nonlinear manifold learning technique as an initial heuristic tool, we extracted a 2-dimensional manifold representing the most important collective properties of the reaction pathways. This manifold was effectively characterized by two functions: reactant proportion (Γ_R.P._) and oxidation degree (Γ_O.D._).

Temperature influence: Higher temperatures accelerate the oxidation reaction, favoring the full oxidation of methane (FOM). Conversely, lower temperatures promote partial oxidation of methane (POM). Interestingly, at *T* = 3500 K, the reaction initially favors POM but transitions to FOM in the final stages.

Impact of the reactant ratio: Fuel-lean and fuel-rich conditions exhibit distinct reaction pathways. As the *n*(CH_4_):*n*(O_2_) ratio increases, the reaction rate also rises. Fuel-lean and stoichiometric conditions predominantly favor FOM, which is characterized by large Γ_O.D._ values throughout the simulation. In contrast, the fuel-rich conditions favor POM and specific reactions (8, 11).

Overall, our research offers useful insights into the initial mechanism of methane oxidation. The application of dMaps analysis provides a clear and systematic interpretation of complex simulation data with potential applications in combustion simulations of other fuels. Future works can be focused on simulating methane oxidation assisted by catalysts under various conditions and applying dMaps to more complex simulations to guide further quantitative analysis, such as the pyrolysis of large hydrocarbons or the oxidation of many-component system. The comparison of the result using DFTB is also of potential interest.

## Supporting Information Available

Full list of the 207 types of molecule species being identified from all simulations and number of main radicals as a function of time for simulation (PDF)
Input files and script for the simulation in Lammps (ZIP)
Typical simulation trajectory video (full 2000ps) (MP4)
Typical simulation trajectory video (0ps) (MP4)
Typical simulation trajectory video (500ps) (MP4)
Typical simulation trajectory video (1950ps) (MP4)
