# Supplementary material for: A Study of the Methane Oxidation Mechanism and Reaction Pathways Using Reactive Molecular Simulation and Nonlinear Manifold Learning
Source: ACS Omega. 2024 Oct 17;9(43):43894–907. doi: 10.1021/acsomega.4c07094 (PMC11525525; doi:10.1021/acsomega.4c07094)
Supplement: Supplementary file 1 — ao4c07094_si_001.pdf [file ao4c07094_si_001.pdf]

SUPPLEMENTARY MATERIAL

A Study of the Methane Oxidation Mechanism and Reaction Pathways Using Reactive Molecular Simulation and Nonlinear Manifold Learning

Jiang Wang,* Jiaxuan Tang, and Fuye Chen

College of Science, Guizhou Institute of Technology, Boshi Road, Dangwu Town, Gui'an New District, Guizhou, 550025, China

E-mail: cwangjiang@git.edu.cn

SUPPLEMENTARY TEXT

From 5 simulations with different conditions, totally 207 different types of molecules and radicals are identified by the LAMMPS built-in algorithm, they form a species basis:

$\mathbb{S} : [s_1, s_2, s_3, \cdots, s_{207}] =$ [CH_4, O_2, CH_4O_2, CH_3, H, HO_2, CH_3O_2, O_4, C_2H_7, CH_5O_2, HO_4, CH_5, C_2H_8, H_2O_2, H_2, CH_2, HO, HO_3, CH_5O, H_2O, C_2H_6, H_3O, O, CH_6, $C_2H_6O_2$, H_2O_4, H_2O_3, CH_6O_2, CH_4O, CH_3O, CH_4O_3, H_3O_3, C_2H_5, C_3H_10, CH_3O_3, CH_2O, CH_6O, $C_2H_7O_2$, C_2H_7O, H_3O_2, CH_2O_3, C_2H_6O, CH_2O_2, C_2H_4, $C_2H_5O_2$, $C_2H_5O_3$, $C_2H_4O_2$, C_2H_5O, CHO, CHO_3, CO, C_3H_10O, C_3H_8, C_2H_3, H_4O_2, $C_2H_3O_2$, C_3H_5O, H_3O_4, C_2H_8O, C_2H_2, CHO_2, CO_2, C_2H_3O, C_3H_7, $C_2H_2O_2$, C_3H_5, $C_3H_5O_2$, H_4O, O_3, C_3H_8O, C_2H_2O, C_2HO, $C_2H_2O_3$, C_2HO_3, H_3, CH_5O_3, C_2H_4O, C_2O, C_2O_3, H_5O_2, CO_3, C_2HO_2, CH, $C_2H_3O_3$, C_3H_9O, CH_2O_4, C_3H_2O, CH_4O_4, CH_3O_4, C_3H_6, C_2H, C_3H_4, C_3H_3, C_3H_4O, C_3H_3O, $C_3H_4O_3$, $C_3H_6O_2$, CHO_4, CO_4, C_3HO_2, H_4O_3, C_2O_2, C, $C_2H_3O_4$, $C_3H_2O_2$, H_5O_3, H_4O_4, C_2O_4, H_6O_3, $C_2H_6O_3$, $C_2H_8O_2$, $C_2H_4O_3$, CH_6O_3, $C_2H_7O_3$, $C_3H_8O_2$, CH_3O_5, CH_5O_4, $C_2H_2O_4$, $C_2H_4O_4$, C_3O_3, H_5O_4, CH_7O_2, H_5O, H_4, CH_2O_5, C_3H_6O, C_3H_9, C_3H_7O, $C_3H_3O_3$, H_6O_2, H_3O_5, O_5, $C_2H_5O_4$, $C_3H_9O_2$, $C_3H_7O_2$, C_4H_8, C_4H_7, $C_3H_4O_2$, C_4H_9, C_5H_9, C_4H_6O, $C_3H_5O_3$, C_4H_5, C_4H_7O, C_3H_2, $C_4H_3O_2$, $C_4H_8O_2$, C_4H_5O, $C_3H_3O_2$, C_4H_3, C_5H_5O, C_4H_3O, C_4H_4, C_5H_8, C_6H_9, C_5H_7O, C_3HO, C_5H_4O, C_3O_2, C_4H_6, C_4H_4O, C_6H_7, C_4H_2O, C_5H_5, C_6H_6, C_8H_9, C_8H_8, C_8H_11O, $C_4H_5O_2$, C_6H_5, C_6H_7O, C_6H_4, C_8H_7, C_11H_11, C_8H_6, C_4H_2, C_5H_3O, C_2, C_5H_7, $C_4H_4O_2$, C_5H_6, C_3O, C_3H, C_5H_6O, C_5H_4, $C_4H_2O_2$, $C_5H_2O_2$, C_4HO, C_5H, C_5, C_5H_2, C_5H_2O, C_6O_2, C_6O, C_5H_3, C_6HO, C_3, C_6H_2O, $C_5H_5O_2$, $C_6H_3O_2$, $C_6H_5O_2$, C_6H_3O, C_4H, C_5HO, C_5HO_2, C_4O, C_4HO_2]

SUPPLEMENTARY FIGURES

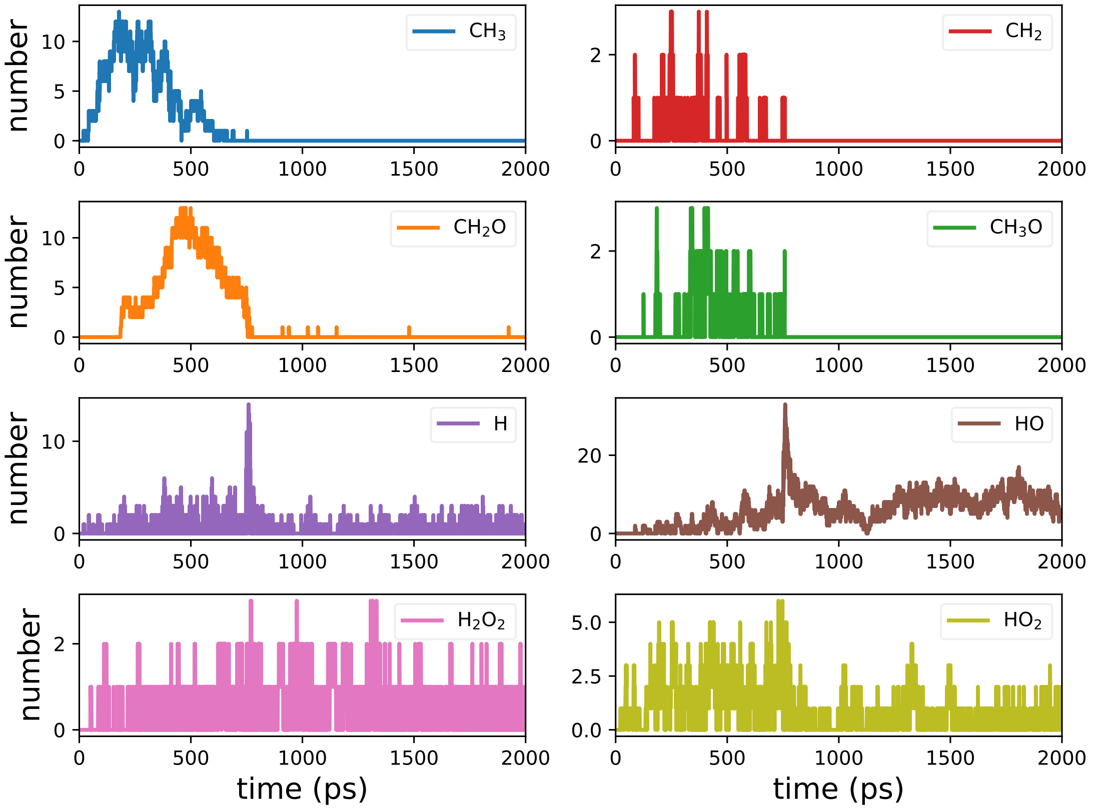

Figure S1: Number of main radicals as a function of time for simulation S0, where T=3500K, L = 3.5 nm, $n(CH_4) : n(O_2) = 1 : 2$.

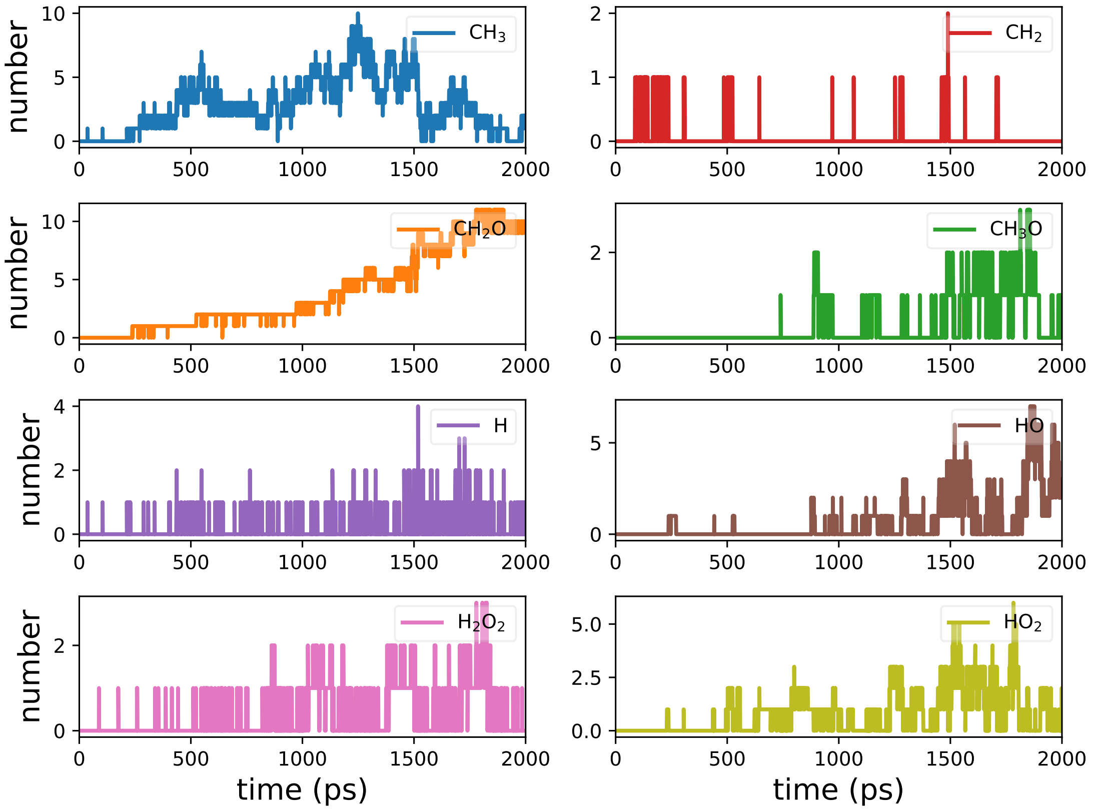

Figure S2: Number of main radicals as a function of time for simulation S1, where T=3000K, L = 3.5 nm, $n(CH_4) : n(O_2) = 1 : 2$.

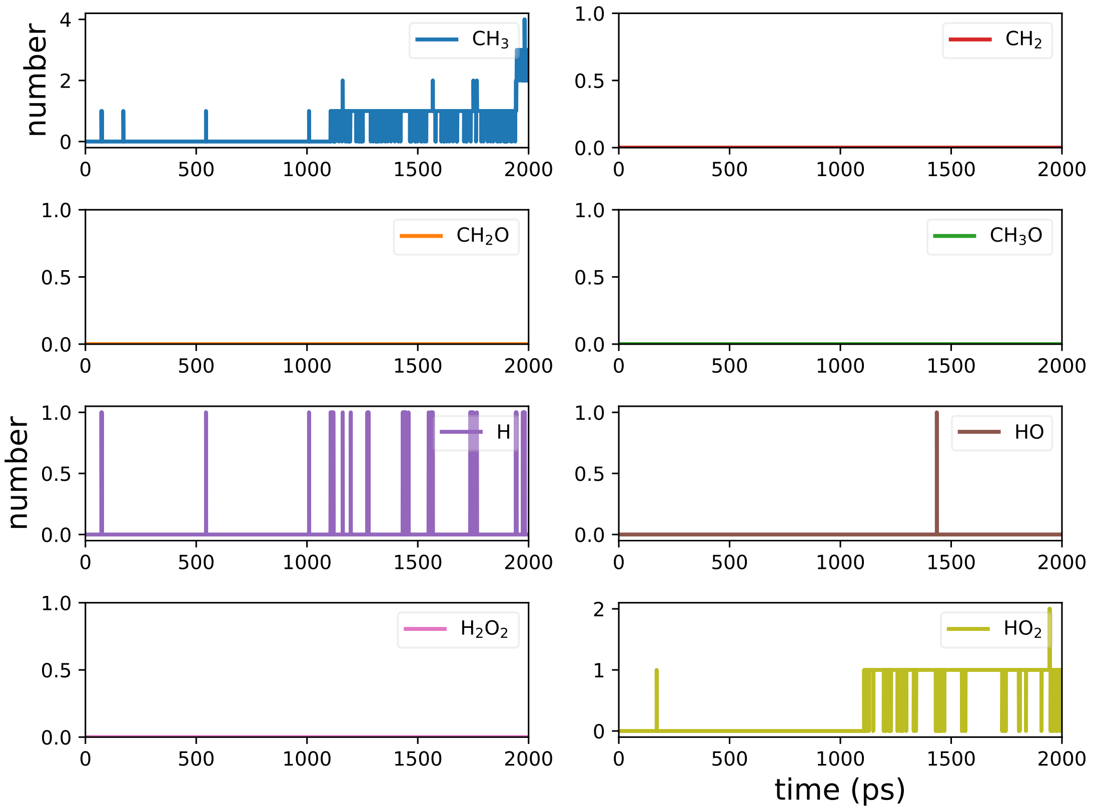

Figure S3: Number of main radicals as a function of time for simulation S2, where T=2600K, L = 3.5 nm, $n(CH_4) : n(O_2) = 1 : 2$.

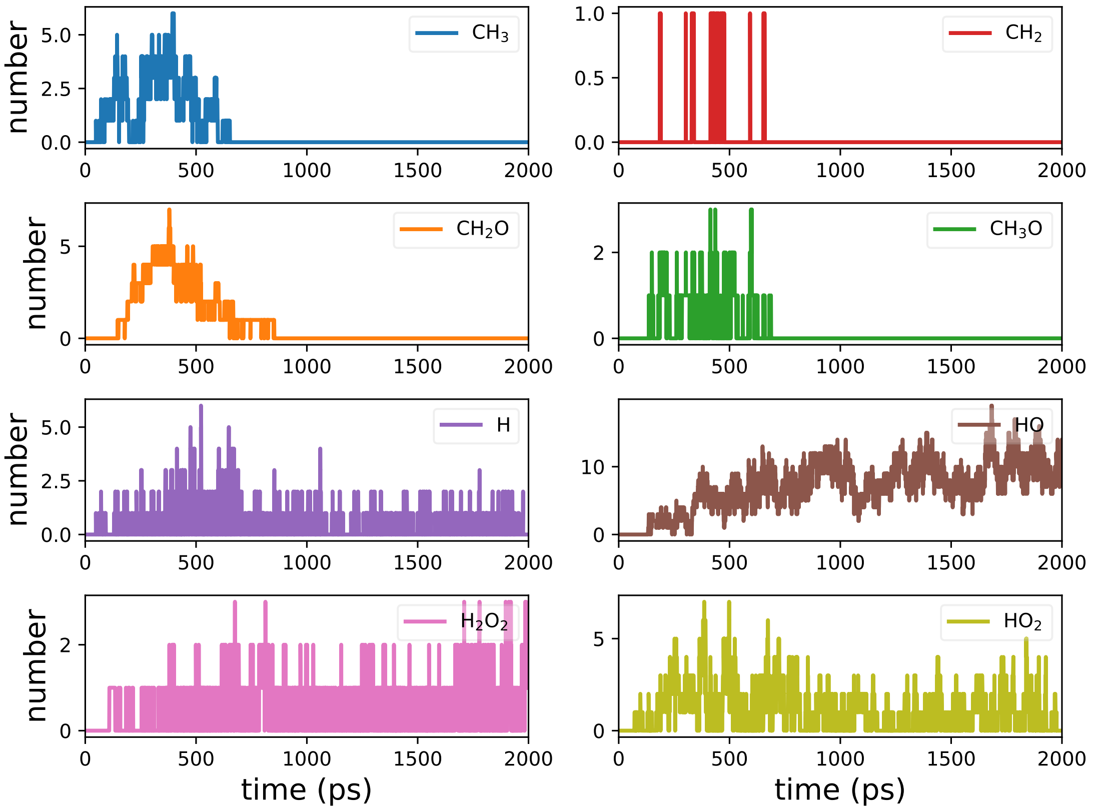

Figure S4: Number of main radicals as a function of time for simulation S3, where T=3500K, L = 3.5 nm, $n(CH_4) : n(O_2) = 1 : 4$.

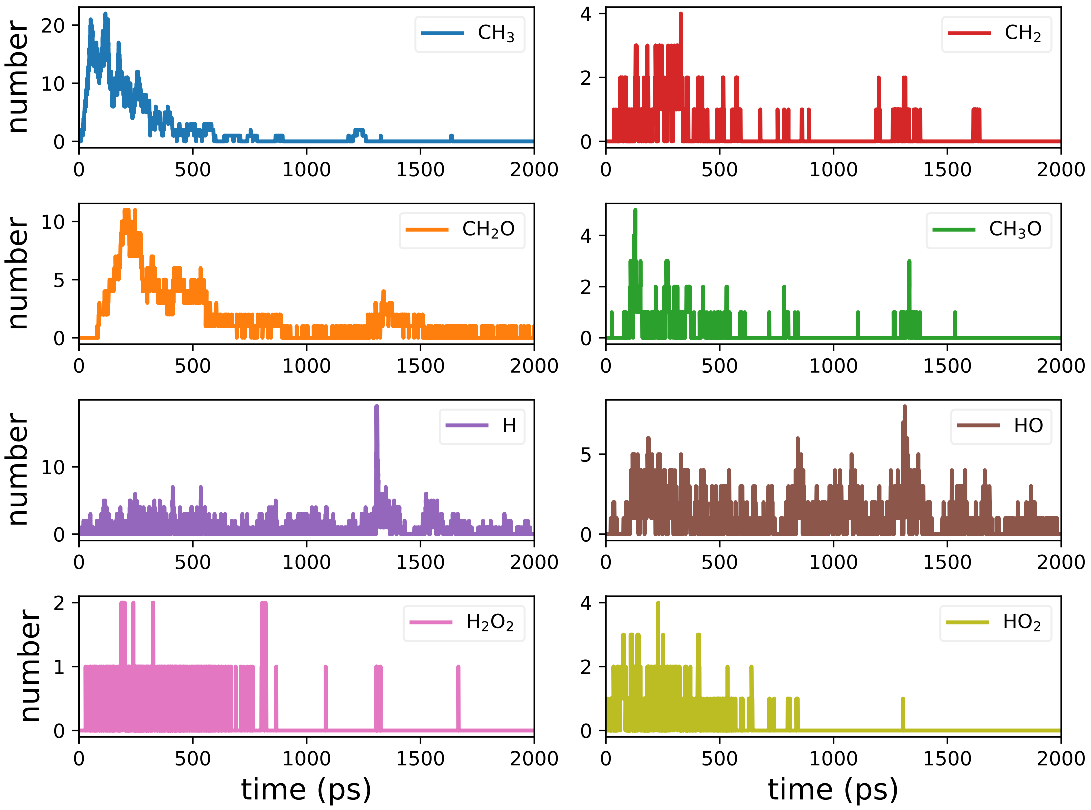

Figure S5: Number of main radicals as a function of time for simulation S4, where T=3500K, L = 3.5 nm, $n(CH_4) : n(O_2) = 3 : 2$.

SUPPLEMENTARY TABLES

Table S1: Reaction potential energy for ReaxFF and DFT of main reactions. The DFT calculations were performed using the Gaussian 09W program. The geometric configurations of the reactants and products were optimized using the B3LYP functional and the 6-31G basis set. The energy difference (ΔE) was calculated by subtracting the potential energy of the reactants from that of the products.

Reactions	R1	R1.2	R2	R3	R4
ΔE ReaxFF (kCal/mol)	101.23	-105.45	101.48	-5.89	-9.45
ΔE DFT (kCal/mol)	79.34	-128.92	139.30	-7.10	-7.29
Relative Error (%)	27.59	18.21	27.15	17.04	29.63

SUPPLEMENTARY VIDEOS

From S0 simulation, four videos of the simulation trajectory are provided:

The file full.mp4 contains the complete 2000 ps simulation trajectory. However, due to the length of the simulation, the fast movement of atoms may not be easily discernible in this video. To provide a clearer visualization, three additional videos displaying slower movement at different time intervals are included: 0ps.mp4, 500ps.mp4, and 1950ps.mp4.
